# A 3D Meso-Scale Model and Numerical Uniaxial Compression Tests on Concrete with the Consideration of the Friction Effect

**DOI:** 10.3390/ma17051204

**Published:** 2024-03-05

**Authors:** Jiawei Wang, Xinlu Yu, Yingqian Fu, Gangyi Zhou

**Affiliations:** 1 Key Laboratory of Impact and Safety Engineering (Ningbo University), Ministry of Education, Ningbo 315211, China; 2211090030@nbu.edu.cn; 2 College of Science and Technology, Ningbo University, Ningbo 315300, China; yuxinlu@nbu.edu.cn; 3 Faculty of Mechanical Engineering and Mechanics, Ningbo University, Ningbo 315211, China; zhougangyi@nbu.edu.cn

**Keywords:** concrete, compression, meso-scale FE, contact friction, failure process

## Abstract

Achieving the real mechanical performance of construction materials is significantly important for the design and engineering of structures. However, previous researchers have shown that contact friction performs an important role in the results of uniaxial compression tests. Strong discreteness generally appears in concrete-like construction materials due to the random distribution of the components. A numerical meso-scale finite-element (FE) method provides the possibility of generating an ideal material with the same component percentages and distribution. Thus, a well-designed meso-FE model was employed to investigate the effect of friction on the mechanical behavior and failure characteristics of concrete under uniaxial compression loading. The results showed that the mechanical behavior and failure profiles of the simulation matched well with the experimental results. Based on this model, the effect of friction was determined by changing the contact friction coefficient from 0.0 to 0.7. It was found that frictional contact had a slight influence on the elastic compressive mechanical behavior of concrete. However, the nonlinear hardening behavior of the stress–strain curves showed a fairly strong relationship with the frictional contact. The final failure profiles of the experiments showed a “sand-glass” shape that might be expected to result from the contact friction. Thus, the numerical meso-scale FE model showed that contact friction had a significant influence on both the mechanical performance and the failure profiles of concrete.

## 1. Introduction

Mechanical performance is deemed a significant feature of most engineering materials used for construction [1,2,3,4]. The uniaxial compression test is one of the simplest and most basic test methods for determining the mechanical properties of concrete-like materials. It is generally expected that the measured values reflect the inherent material properties. However, the mechanical response is affected by various parameters, including specifics regarding loading by the machine, measurement devices, the geometric shape of the specimen, the loading rate, boundary conditions, etc. [5,6,7,8,9]. Especially for brittle materials, the influence of the boundary conditions has a pronounced effect on the mechanical performance of the quickly softening regime caused by the evolution of the inherent damage [10,11,12,13,14].

In most previous studies, concrete is usually assumed to be a homogeneous material. In fact, concrete is a kind of heterogeneous composite material consisting of coarse aggregates, a mortar matrix, an interface transition zone (ITZ), and voids [15,16]; concrete’s macro-scale strength and failure must be related to the mechanical performance of these components at a meso- or micro-scale. A meso-finite-element model provides the possibility to bridge the meso-scale behavior of components and the macro-scale response of structures [17,18,19]. The components are usually distributed randomly in the concrete, which makes the experimental results unrepeatable. The numerical meso-scale finite-element (FE) method provides the possibility of generating an ideal material with the same component percentages and distribution. Zhang [20,21] constructed a meso-finite-element model to simulate uniaxial compressive tests on concrete while considering the connecting friction. The results showed that the frictional constraint had a strong influence on the peak stress value and the softening phase. When the frictional coefficient increased from 0.1 to 0.3, the peak stress could have a significant increase of up to 126%.

Previous researchers have found the sensitivity of the experimental results to the friction between an upper or lower rigid platen and the connected surfaces of cubes. Bandeira [22] investigated the influence of the boundary conditions and geometries of specimens through unconfined compression tests on concrete. Three anti-friction strategies were carried out on the loaded planes: grease, Teflon, and brush plates. The reported results proved that post-peak softening is not a characteristic of the materials, but a consequence of the interface friction in the tests. Torrenti [23] conducted serious uniaxial compressive tests to investigate the influence of friction and found that friction has a strong influence on the failure profiles of concrete. Cubic specimens would completely fracture and result in the immediate loss of bearing capacity as soon as the maximum load was reached when the connecting friction was eliminated. In this manner, the friction at the boundaries is responsible for considerable influence on not only the compressive strength, but also the failure profiles.

Although the effects of friction have been the focus of previous research, the macro-mechanical behavior of concrete and the meso-scale local failure mechanism of the components have not been well investigated with the consideration of the changes to the friction at the boundaries in uniaxial compression tests. In this paper, a well-designed 3D-meso FE model of concrete was constructed to investigate the effect of friction on the mechanical response and failure behavior. This simulation work was conducted to show the uniaxial compression damage process and analyze crack evolution to explore the mechanism of macro-destruction and discuss the influence of boundary friction on the damage, as well as the mechanical properties of concrete.

## 2. Methodology

### 2.1. Experimental Programs

#### 2.1.1. Materials and Specimens

In this study, ordinary Portland cement (P. I. 42.5) with a 28-day compression strength of 42.5 MPa was used. The coarse aggregates were round in shape and had a size ranging from about 3 mm to 12 mm. The fine aggregate was composed of river sand with a specific gravity of 2.6. The ratio of water to cement was 0.5.

The mixes were prepared and cured under laboratory conditions. All concrete cubes had a size of 50 mm × 50 mm × 50 mm and were cast simultaneously and cured for 28 days under the same ambient conditions (20 ± 2${\;}^{{}^{\circ}}$C and 95% relative humidity).

#### 2.1.2. Experimental Setup

**Figure 1:**
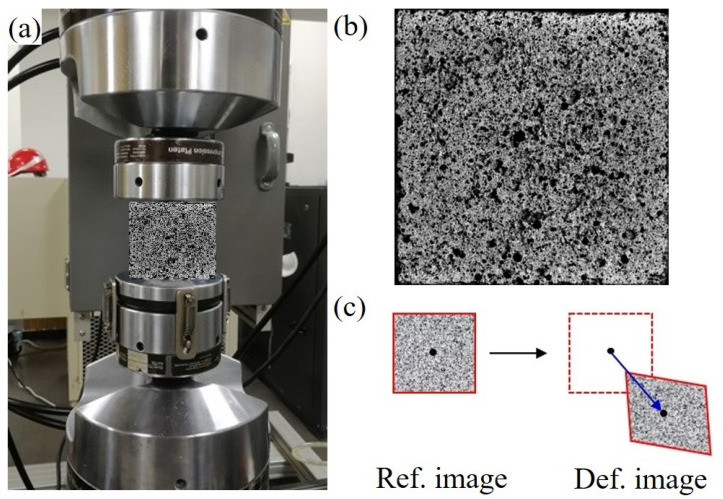
Experimental setups of uniaxial compression using an MTS machine (**a**) to load the specimens with speckles (**b**) spread on the surface of cubes calculated using the DIC method (**c**).

Figure 1 shows the specific setup for the uniaxial compressive tests of the concrete. A hydraulic servo material testing machine (MTS-180-50; shown in Figure 1a) with a constant compressive velocity of 0.5 mm/s was utilized to load concrete cubic specimens under a quasi-static condition. To accurately measure the micro-strain of the brittle materials, the digital image correlation (DIC) technique was employed to measure the real-time strain by spraying speckles on the surface of the cubes, as shown in Figure 1b. To capture the evolution of cracking, a Photron FASTCAM SA.5 camera was adopted to record the surface deformation of the specimens. Then, the DIC technique was applied to detect the initiation of cracks by measuring the strain concentration in real time. The relative motion of the region of interest (ROI) could be tracked by comparing the grey-level distributions of the reference and deformed images. An accurate strain and cracking process for concrete can thus be determined using the DIC method and a high-resolution camera.

### 2.2. Meso-Scale Finite-Element Model

#### 2.2.1. Geometry of Coarse Aggregates

**Figure 2:**
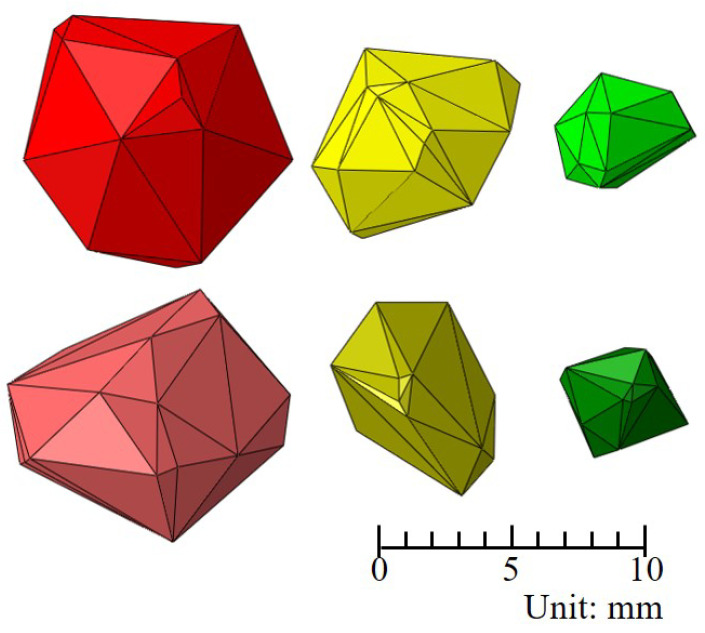
Several typical coarse aggregates used in this model.

Herein, the 3D Voronoi diagram method was used to generate coarse aggregates with a specific size distribution. Coarse aggregates were randomly distributed inside a concrete cube with a size of 50 mm × 50 mm × 50 mm to simulate the cubic specimen fabricated in a mold. Figure 2 shows six typical polyhedrons generated using the 3D Voronoi diagram method, closely resembling the shapes and sizes of the actual aggregates utilized in the experiment.

#### 2.2.2. Grading of Coarse Aggregate

The size distribution of coarse aggregates is typically determined by analyzing a grading curve. A grading curve depicts the cumulative percentage passing through sieve openings of various sizes. Among the methods commonly employed to describe this distribution for typical concrete aggregates is the Fuller curve, which can be expressed as:

(1) $$P\left(d\right)=100{\left(\frac{d}{d_{max}}\right)}^{n}$$

where P(d) is the corresponding passing percentage (%), *d* is the diameter of each grading class, and $d_{max}$ is the maximum aggregate diameter. In this study, the maximum size, $d_{max}$, was 12.7 mm, and *n* is an exponent of the chosen grading curve. Generally, *n* is taken as 0.5, which was used in this model.

**Figure 3:**
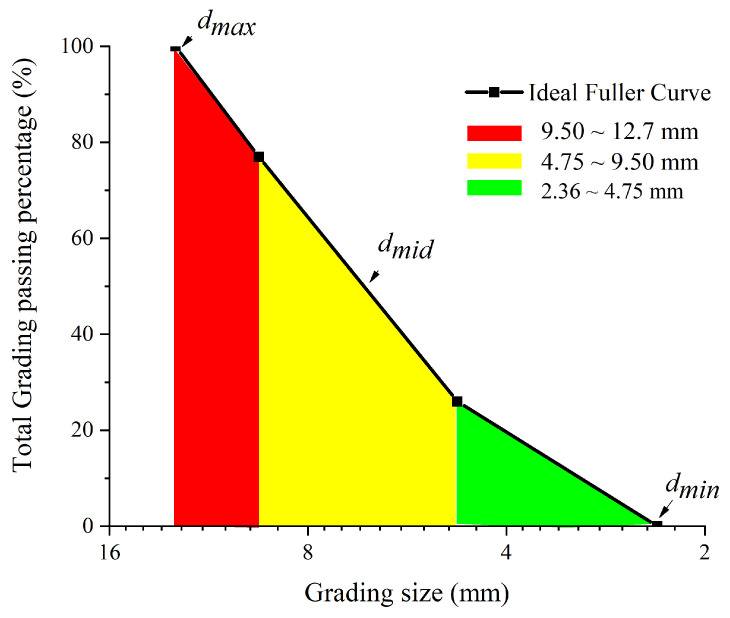
Total grading passing percentage of aggregates used in this model.

In the meso-FE model, the grain size distribution is designed as a classic Fuller curve with a size ranging from about 2.36 mm to 12.7 mm [24]. The size distribution of aggregates is shown in Table 1, which is very close to that of the experimental matrix with a size ranging from 2.36 mm to 12.7 mm. The total grading passing percentage of aggregates is described in Figure 3. For sizes of less than 2.36 mm, the coarse and fine aggregates were considered as mortar for their increasing computational efficiency.

#### 2.2.3. Amount of Coarse Aggregate

After the size distribution of aggregate particles is given as the Fuller grading curves, then the amount of aggregates within the grading segment $\left[d_{i},d_{i+1}\right]$ can be calculated as:

(2) $$N\left[d_{i},d_{i+1}\right]=\frac{V_{P}\left[d_{i},d_{i+1}\right]}{V_{e}}$$

where $V_{e}$ is the equivalent volume of a single aggregate in the range $\left[d_{i},d_{i+1}\right]$. Generally, $V_{e}$ was calculated according to $\frac{4}{3}\pi d_{med}^{3}$, where $d_{med}$ was the median diameter of $\left[d_{i},d_{i+1}\right]$, where $V_{e}\left[d_{i},d_{i+1}\right]$ can be expressed as:

(3) $$V_{p}\left[d_{i},d_{i+1}\right]=\frac{P\left(d_{i}\right)-P\left(d_{i+1}\right)}{P\left(d_{max}\right)-P\left(d_{min}\right)}\times v_{p}\times V$$

where $d_{max}$ and $d_{min}$ are the maximum and minimum size of aggregates, namely 12.7 mm and 2.36 mm respectively; $v_{p}$ is the volume fraction of aggregates, and *V* is the volume of the concrete sample.

The volume fraction of aggregate $v_{p}$ of the concrete can be determined as:

(4) $$v_{p}=\frac{w_{p}}{\rho_{p}V}$$

where $V_{p}$ is the volume fraction of aggregates, $w_{p}$ is the total weight of aggregate particles, $\rho_{p}$ is the specific weight, and *V* is the total volume of the specimen.

In normal-strength concrete, the total volume of aggregates is generally taken to be no greater than 70% of the entire volume (*V*), while the volume of coarse aggregates is expected to be up to 40% of *V* [25]. Therefore, the volumetric fraction of coarse aggregates assumed as 30% seemed reasonable for the meso-scale FE model.

According to the size distribution of aggregates in Table 1, the grain number of aggregates calculated as Equation (2) is shown in Table 2. In this meso-scale model, 344 aggregates were generated randomly according to a classic Fuller curve.

**Figure 4:**
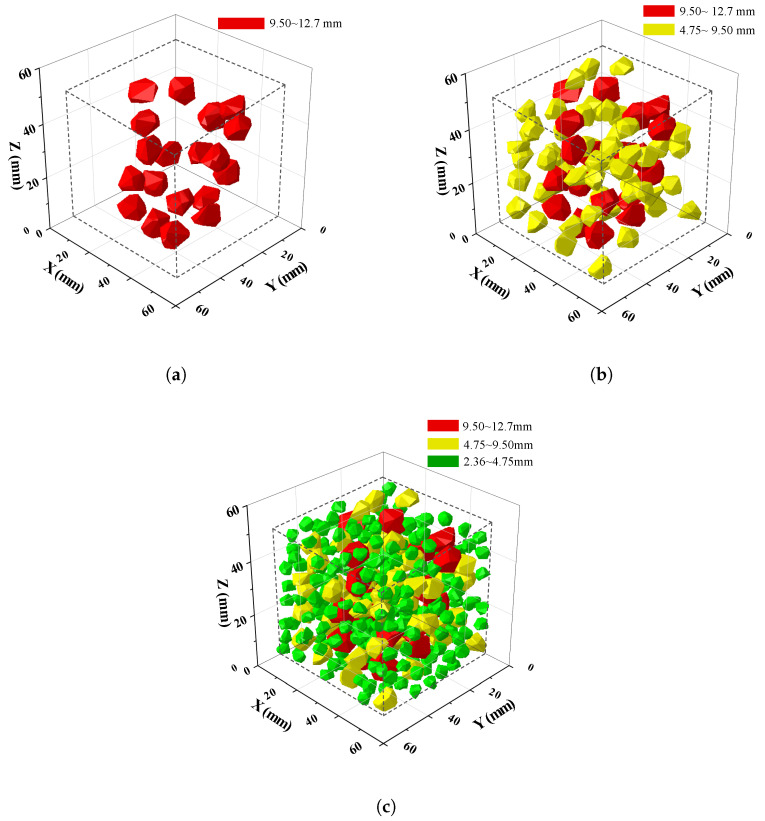
Insertion of aggregates in the cube with three different grading sizes. (**a**) Grading from 9.50 mm to 12.7 mm, (**b**) grading from 4.75 mm to 12.7 mm, and (**c**) grading from 2.36 mm to 12.7 mm.

Figure 4 shows the spatial distribution of aggregates in a 50 mm × 50 mm × 50 mm cube with three different grading sizes ranging from 2.36 mm to 12.7 mm. It can be observed that coarse aggregates are randomly placed in the cube, guaranteeing no intersection between any two of them.

#### 2.2.4. ITZ Layer

Generally, primary macro-cracks originate at weakened interfaces or within the inner voids between the mortar matrix and coarse aggregates. Discrete cracks often exhibit pronounced curvature along aggregate grains, but seldom propagate through a single weak aggregate grain. Therefore, a three-phase meso-scale model, including a mortar matrix, aggregates, and an ITZ, was most widely employed in previous meso-FE numerical studies on concrete. An ITZ was observed to possess a layered structure characterized by lower density and reduced mechanical strength in comparison to the surrounding mortar matrix. Skarzynski [26] et al. measured the width of ITZs using a scanning electron microscope with a magnification factor of 30,000, and found that the width of ITZ layers along the aggregate particles was arranged from 30 to 50 μm.

**Figure 5:**
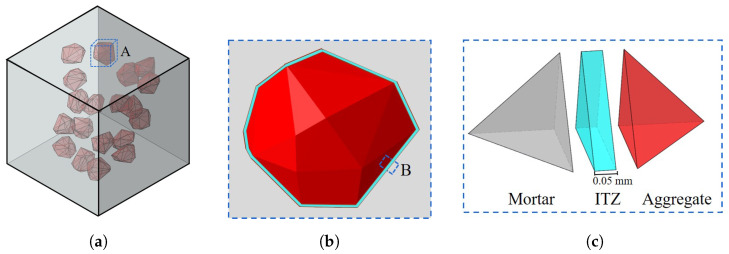
The three-phase structure of the concrete employed in the meso-scale FE model. (**a**) Macro-scale model of the cube, (**b**) three phases of Zone A, and (**c**) elements of Zone B in the three-phase model.

Figure 5 gives the finite-element model of concrete with three phases including mortar (color of gray element), aggregate (color of red element), and ITZ (color of blue element). The cohesive element was employed to describe the ITZ layer elementsandwiched between the mortar element and coarse aggregate element. To avoid the generation of unacceptable numerous elements in the FE model, the thickness of the ITZ was set at 0.05 mm, a dimension closely approximating the actual layer thickness, as depicted in Figure 5c.

### 2.3. Material Properties

The meso-scale mechanical behavior of concrete was taken as relatively complex due to a random array of morphological features inherent in the concrete microstructure. In meso-scale FE simulation, the aggregates were assumed as a linear elastic material without consideration of the nonlinear behavior, damage, or cracking. For the mortar, the concrete damage plasticity (CDP) model was employed for the wide use range of its constitutive laws in meso-scale analyses of concrete [21,27]. A cohesive element with damage evolution was adopted to identify the inter-phase crack nucleation and propagation. The detailed constitutions of the phases in concrete are described as follows.

#### 2.3.1. The CDP Model

**Figure 6:**
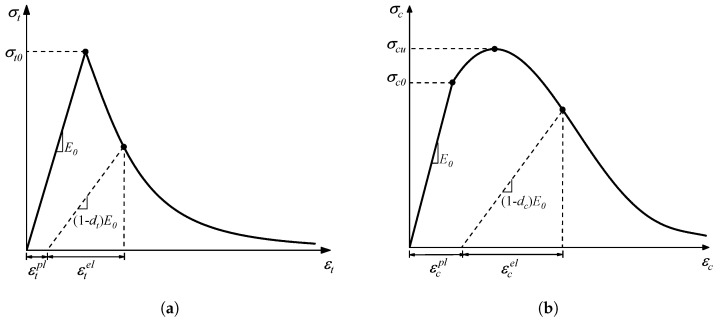
The response of concrete to uniaxial loading in the CDP model. (**a**) Tensile cracking and (**b**) compressive crushing.

The CDP model is a continuum, plasticity-based damage model of concrete [28]. There are two main failure mechanisms assumed in this model, namely (1) tensile cracking and (2) compressive crushing. Under uniaxial tension loading, the stress–strain response followed a linear elastic relationship before the onset of tensile cracking. Once the load reached the failure stress, $\sigma_{tf}$, it began to form, leading to softening in the stress–strain curve at a macroscopic level. Under uniaxial compressive loading, the stress–strain response exhibited linearity up to the yield stress, $\sigma_{cy}$. Subsequently, there was a phase of relatively weakened plastic hardening before the stress–strain response transitioned into softening beyond the ultimate stress, $\sigma_{cf}$. The typical stress–stain curves of tensile crackingand compressive crushing are shown in Figure 6. The stress–strain relations at the failure stage are governed by scalar damaged elasticity, as shown in Equation (5).

(5) $$\sigma =\left(1-D\right)E_{o}^{el}\left(\varepsilon -\varepsilon^{pl}\right)=E^{el}\left(\varepsilon -\varepsilon^{pl}\right),0\leq D\leq 1$$

where σ is the stress. ε and $\varepsilon^{pl}$ represent the strain and the plastic strain. $E_{0}^{el}$ is the initial (undamaged) elastic modulus of the materials. $E^{el}$ = $\left(1-D\right)E_{0}^{el}$ is the degraded elastic modulus. *D* is the scalar stiffness degradation variable, where 0 means the undamaged state and 1 means the fully damaged stage.

A fracture energy cracking criterion is employed to describe the progressing tensile cracking of concrete, where the fracture energy, $G_{f}$, is defined as the energy required to open a unit area of the crack. The cracking displacement $u_{t0}$ at which a complete loss of strength took place is expressed as Equation (6):

(6) $$u_{t0}=\frac{2G_{f}}{\sigma_{t0}}$$

where $\sigma_{t0}$ is the tension strength. The typical value of $G_{f}$ is taken as 120 N/m for concrete with a compressive strength of 40 MPa.

The yield functions expressed by effective stress, $\bar{\sigma }$, and effective plastic strain, ${\bar{\varepsilon }}^{pl}$, are presented as Equation (7).

(7) $$f\left(\bar{\sigma },{\tilde{\varepsilon }}^{pl}\right)=\frac{1}{1-\alpha }\left(\bar{q}-3\alpha \bar{p}+\beta \left({\tilde{\varepsilon }}^{pl}\right)\langle {\hat{\bar{\sigma }}}_{max}\rangle -\gamma \langle -{\hat{\bar{\sigma }}}_{max}\rangle \right)-{\bar{\sigma }}_{c}\left({\tilde{\varepsilon }}_{c}^{pl}\right)$$

where $\bar{p}$ is the effective hydro-static pressure. $\bar{q}$ is the Mises equivalent effective stress. ${\hat{\bar{\sigma }}}_{max}$ is the algebraically maximum eigenvalue of effective stress, $\bar{\sigma }$.

The function of $\beta \left({\tilde{\varepsilon }}^{pl}\right)$ is expressed as:

(8) $$\beta \left({\tilde{\varepsilon }}^{pl}\right)=\frac{{\bar{\sigma }}_{c}\left({\tilde{\varepsilon }}_{c}^{pl}\right)}{{\bar{\sigma }}_{t}\left({\tilde{\varepsilon }}_{t}^{pl}\right)}\left(1-\alpha \right)-\left(1+\alpha \right)$$

where ${\bar{\sigma }}_{t}$ and ${\bar{\sigma }}_{c}$ are the effective tensile and compressive cohesion stress, respectively.

α and γ are dimensionless material constants. The constant of α is expressed by Equation (9):

(9) $$\alpha =\frac{\left(\sigma_{b0}/\sigma_{c0}\right)-1}{2\left(\sigma_{b0}/\sigma_{c0}\right)-1};\left(0\leq \alpha \leq 0.5\right)$$

where $\sigma_{b0}$ and $\sigma_{c0}$ represent the initial equibiaxial and uniaxial compressive yield stress. Lubliner et al. [28] gave a typical experimental value of the ratio $\frac{\sigma_{b0}}{\sigma_{c0}}$ for concrete ranging from 1.10 to 1.16.

The constant of γ is expressed by Equation (10).

(10) $$\gamma =\frac{3\left(1-K_{c}\right)}{2K_{c}-1}$$

where $K_{c}$ is the ratio of the second stress invariant of the tensile meridian, $q_{\left(TM\right)}$, to that of the compressive meridian, $q_{\left(CM\right)}$, at the initial yield of any given value of the pressure invariant *p* so that the maximum principal stress is negative. It must satisfy the condition 0.5 < $K_{c}$ ≤ 1.0, where the default value is $\frac{2}{3}$ for the concrete.

Moreover, a non-associated potential plastic flow is assumed based on the CDP model. The flow potential *G* used for this model is the Drucker–Prager hyperbolic function expressed as Equation (11):

(11) $$G=\sqrt{{\left(\in \sigma_{t0}\tan\psi \right)}^{2}+{\bar{q}}^{2}}-\bar{p}\tan\psi$$

where ψ is the dilation angle measured in the *p*-*q* plane at a high confining pressure, whose value was taken by default as 30°. $\sigma_{t0}$ is the uniaxial tensile stress at failure. ∈ is a parameter, referred to as the eccentricity of materials, which was set as ∈ = 0.1 for the concrete in this study.

#### 2.3.2. Cohesive Elements of ITZ

Generally, the onset of microcracks in concrete was thought to occur first in ITZ layers. In order to simulate the fracture process, the ITZ layers were replaced by cohesive elements for describing cracks as jumps in a displacement field. The elastic behavior of cohesive elements is governed by Equation (12):

(12) $$t=\left\{\begin{array}{c} t_{n}\\ t_{s}\\ t_{t} \end{array}\right\}=\left[\begin{array}{ccc} K_{nn} & K_{ns} & K_{nt}\\ K_{ns} & K_{ss} & K_{st}\\ K_{nt} & K_{st} & K_{tt} \end{array}\right]\left\{\begin{array}{c} \varepsilon_{n}\\ \varepsilon_{s}\\ \varepsilon_{t} \end{array}\right\}=K\varepsilon$$

where t is a nominal traction stress vector consisting of three components: the normal traction of $t_{n}$ and two shear tractions of $t_{s}$ and $t_{t}$. K is the stiffness matrix, where $K_{nn}$, $K_{ns}$, *…*, $K_{tt}$ are the components of K. ε is the nominal strain vector, where it can be expressed by the components of $\varepsilon_{n}$, $\varepsilon_{s}$, and $\varepsilon_{t}$. The component of nominal strains can be expressed by the corresponding separations $\delta_{n}$, $\delta_{s}$, and $\delta_{t}$, as shown in Equation (13):

(13) $$\varepsilon_{n}=\frac{\delta_{n}}{T_{o}},\varepsilon_{s}=\frac{\delta_{s}}{T_{o}},\varepsilon_{t}=\frac{\delta_{t}}{T_{o}}$$

where $T_{0}$ is the original thickness of the cohesive element, which was set as 0.05 mm in this study.

For a traction separation model, the stiffness of the interface relating the nominal traction stress to the separation displacement can be carried over to a cohesive layer of the initial thickness, $T_{0}$, as described in Equation (14).

(14) $$K^{\prime }=\frac{K}{T_{0}}$$

Without considering the couple behavior between the normal and shear components, the off-diagonal terms in the elasticity matrix, K, can be set as zero. Thus, the elastic behavior of cohesive elements can be rewritten as Equation (15):

(15) $$t=\left\{\begin{array}{c} t_{n}\\ t_{s}\\ t_{t} \end{array}\right\}=\left[\begin{array}{ccc} K_{nn}^{\prime } & 0 & 0\\ 0 & K_{ss}^{\prime } & 0\\ 0 & 0 & K_{tt}^{\prime } \end{array}\right]\left\{\begin{array}{c} \delta_{n}\\ \delta_{s}\\ \delta_{t} \end{array}\right\}=K^{\prime }\delta$$

where $K_{nn}^{\prime }$ = $K_{nn}$/$T_{0}$, $K_{ss}^{\prime }$ = $K_{ss}$/$T_{0}$, $K_{tt}^{\prime }$ = $K_{tt}$/$T_{0}$. It is recommended to assume the following stiffness of the cohesive elements for the concrete–concrete interface:

(16) $$K_{nn}^{\prime }\approx \frac{E_{c}}{T_{0}}\;K_{ss}^{\prime }=K_{tt}^{\prime }\approx \frac{G_{c}}{T_{0}}$$

where $E_{c}$ and $G_{c}$ are the Young’s and Kirchhoff’s modules of the weaker concrete, respectively. Here, $E_{c}$ and $G_{c}$ were set to be the same as those of the mortar. Generally, a weakening ratio factor was assumed to describe the ratio of the material strength between the ITZ and mortar. The weakened ratio factor was arranged from 0.5 to 0.9, the same as that of previous meso-scale numerical models of concrete. In this study, the ratio was set as 70% [21,29], which is acceptable for a general range.

In this work, a quadratic nominal stress criterion was set to describe the damage initiation of cohesive elements. When the nominal stress ratio reaches 1, the damage is assumed to initiate as expressed by Equation (17):

(17) $${\left\{\frac{\langle t_{n}\rangle }{t_{n}^{o}}\right\}}^{2}+{\left\{\frac{t_{s}}{t_{s}^{o}}\right\}}^{2}+{\left\{\frac{t_{t}}{t_{t}^{o}}\right\}}^{2}=1$$

where $t_{n}^{o}$, $t_{s}^{o}$, and $t_{t}^{o}$ represent the critical traction in fracture mode *I* and fracture mode II along the first and second pure shear direction. In this study, $t_{n}^{o}$ and $t_{s}^{o}$ = $t_{t}^{o}$ were taken as the tensile strength and shear strength of the ITZ layers, respectively. Here, 〈〉 is the Macaulay bracket:

(18) $$\langle t_{n}\rangle =\left\{\begin{array}{cc} 0, & t_{n}<0\\ t_{n}^{o}, & t_{n}\geq 0 \end{array}\right.$$

**Figure 7:**
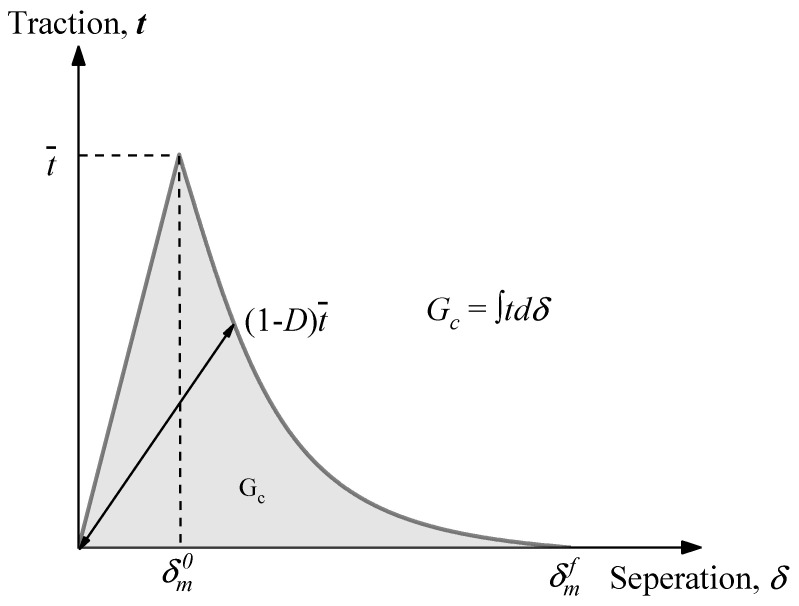
Exponential damage evolution of ITZ layer.

To describe the evolution of damage, it is useful to introduce a fracture energy $G_{c}$ of ITZ. The exponential softening curve was chosen to describe the behavior of cohesive elements in a post-peak regime as shown in Figure 7. The scalar damage variable *D*, dependent on the effective relative displacement, was calculated as:

(19) $$D=\int_{\delta_{m}^{o}}^{\delta_{m}^{f}}\frac{T_{eff}d\delta }{G_{c}-G_{o}}$$

where $T_{eff}$ is the effective traction, $T_{eff}$= $\sqrt{t_{n}^{2}+t_{s}^{2}+t_{t}^{2}}$. $G_{o}$ is the elastic energy at damage initiation.

#### 2.3.3. The Constitutive Parameters

In summary, the meso-scale FE model of concrete employed three constitutive models, each corresponding to a specific phase. Coarse aggregate elements were modeled using a linearly elastic model. The mortar phase utilized the CDP model, while the interface behavior of the ITZ layer between the coarse aggregate and mortar matrix was described using the cohesive crack model. Table 3 provides the constitutive parameters used in these models.

### 2.4. Mesh Generation and Convergence

**Figure 8:**
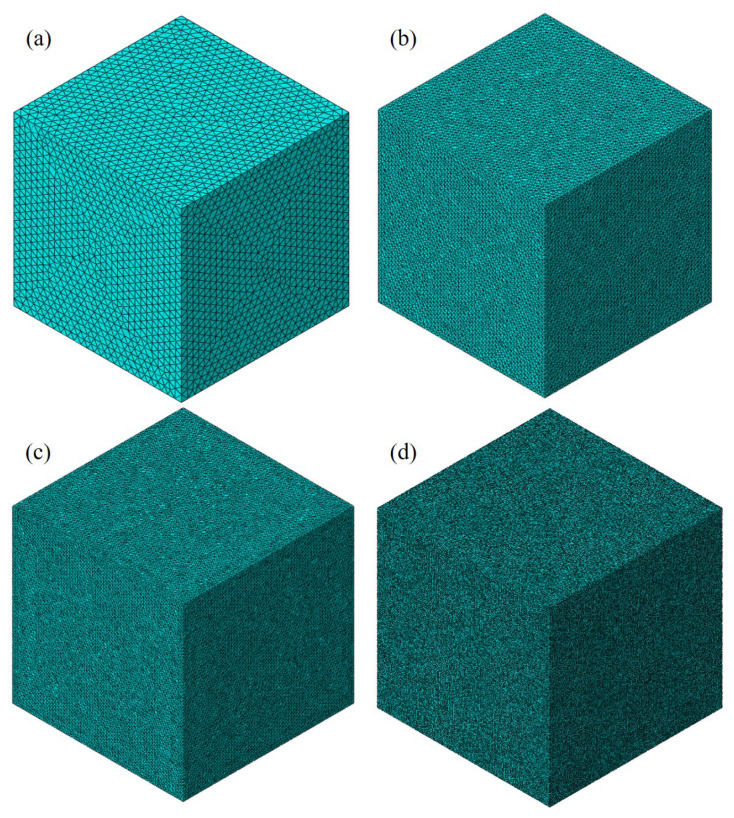
The mesh size of (**a**) 2 mm, (**b**) 1 mm, (**c**) 0.5 mm, and (**d**) 0.1 mm.

**Figure 9:**
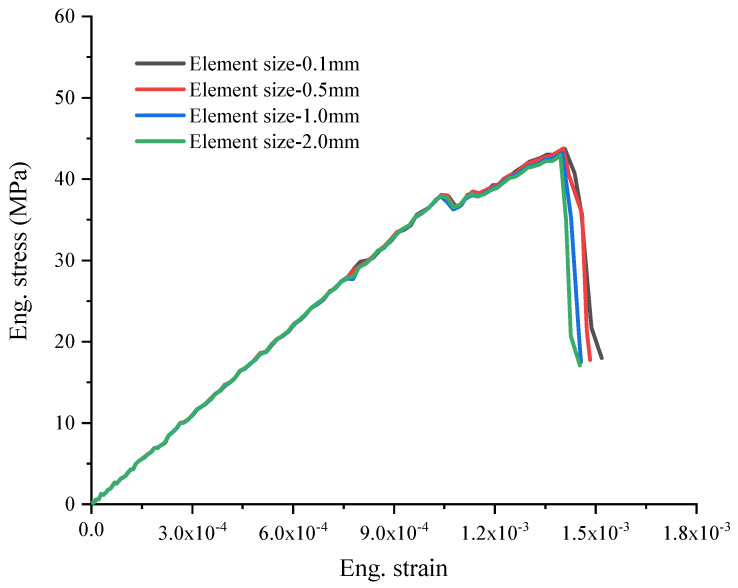
The engineering stress–strain curves with different element sizes.

**Figure 10:**
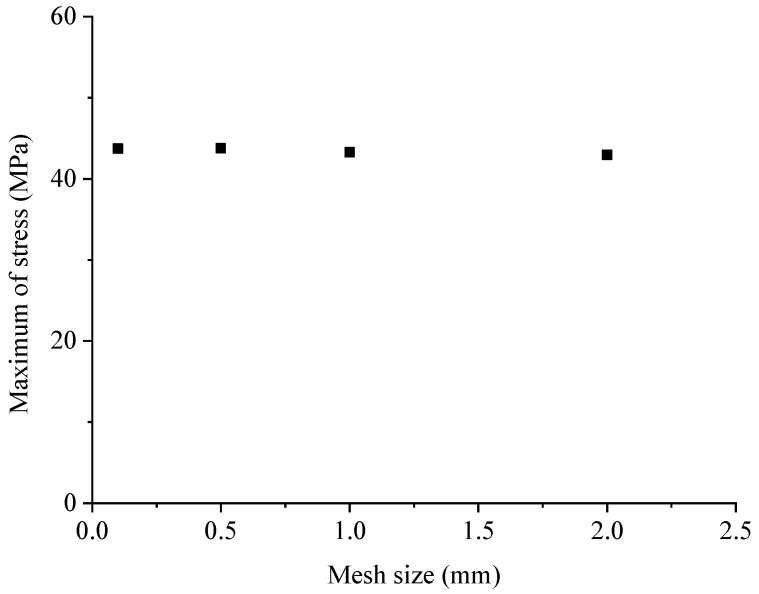
The mesh convergence using four different mesh configurations with an average element size of 0.1 mm–2 mm.

The mesh-generation process was hereby divided into two steps: In the first step, the initial mesh of tetrahedron elements without cohesive elements was defined using Abaqus. In the second step, the cohesive elements were placed between the solid elements of the mortar matrix and aggregates. The mesh convergence was discussed using four mesh sizes arranged from 0.1 mm to 2 mm (Figure 8), which was the average length of the elements. Figure 9 provides the engineering stress–strain curves with different element sizes. While minor variations in mesh configurations might result in slight changes in the numerical stress–strain curves, the engineering stress–strain curves remained fairly comparable until the stress approached its maximum. The significant disparity in the stress–strain curves typically occurred during the post-failure stage. Although slightly different meshes could lead to a slight change in the numerical stress–strain curves, the engineering stress–strain curves were quite comparable before the stress reached the maximum. The main difference of the stress–strain curves was reflected in the post-failure stage. Figure 10 shows the maximum compressive stress with different mesh sizes, where a quite close maximum compressive stress can be clearly observed. From the result, it was deduced that the mesh dependence was negligible for the selected element sizes before the loss of strength. Thus, an element length of 1 mm was selected in this study, though finer meshes would lead to a high computational cost. The element information of components is shown in Table 4 in detail.

### 2.5. Boundary and Load Conditions

**Figure 11:**
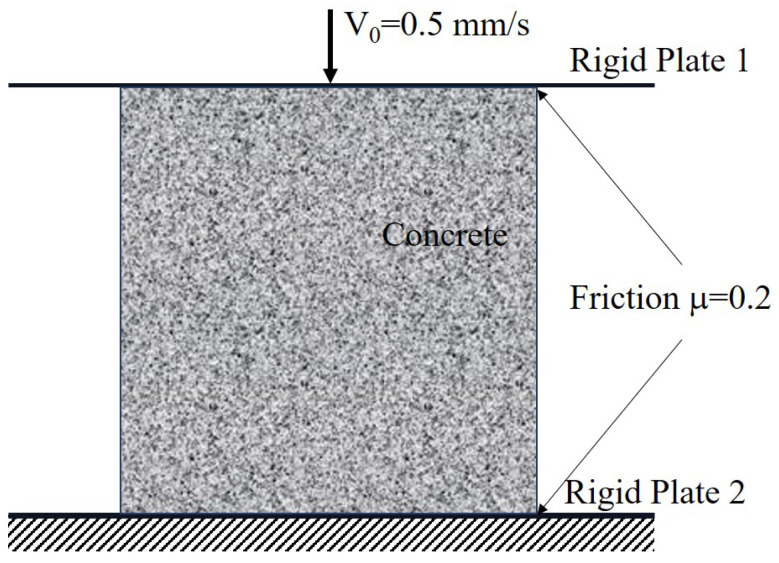
The boundary and loading conditions with the consideration of the contacting friction.

A numerical compression test was carried out with the consideration of the contacting friction between the rigid plate and the concrete cube, as shown in Figure 11. The friction factor was taken as μ = 0.2, which represents the boundary condition of static friction. In this study, all freedoms of the bottom plate (Rigid Plate 2) were constrained. In the vertical direction, a displacement boundary condition was allowed with a constant velocity of 0.5 mm/s for the upper plate (Rigid Plate 1), where other freedoms were constrained.

## 3. Results

In this section, meso-scale FEM simulations were performed using the commercial FE software Abaqus 6.12/Explicit [30] with a displacement condition in the vertical direction applied to the upper rigid plate in the compressive tests. The experimental results of the strain and fractures were exhibited with the field on the surface of the specimens calculated using the DIC method.

### 3.1. Mechanical Behaviors

#### 3.1.1. Linear Elastic Behaviors

**Figure 12:**
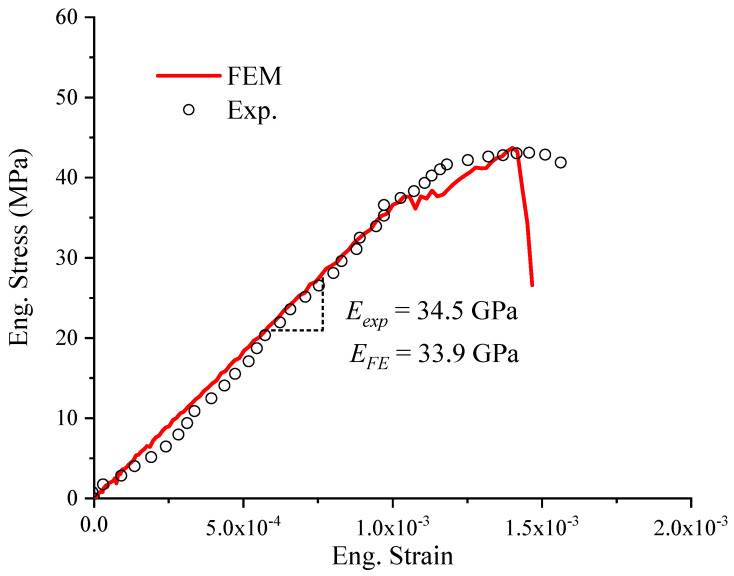
The stress–strain curves obtained from the experimental and numerical results.

The stress–strain curves obtained from the compressive experiment and simulation are shown in Figure 12, where the experimental strain was calculated based on the DIC in the vertical direction. The engineering stress and engineering strainexpressed as Equation (20) were used in Figure 12. It was found that the mechanical behavior of the simulation matched well with the experimental results at the stage of elasticity. The two elastic modules were quite close (error ≤ 1.7%), which were 33.9 GPa and 34.5 GPa from the numerical and experimental results, respectively.

(20) $$\left\{\begin{array}{c} \sigma =\frac{P}{A_{0}}\\ \varepsilon =\frac{\Delta L}{L_{0}} \end{array}\right.$$

where *P* is the load of the rigid plate; $A_{0}$ is the initial cross-area of the cube; ΔL is the dimension of the compression deformation; $L_{0}$ is the initial height of the cube.

In composite materials, the elastic properties can be approximated using a simple homogeneous theory based on the Mori–Tanaka method and the generalized self-consistent method. This involves calculating the weighted average of the modulus of individual components [31,32,33]. Using this approach, the unknown mechanical properties at the macro-scale could be theoretically calculated using the known mechanical properties of each constituent phase at the meso-scale in the range of linear elasticity. Herein, the ITZ layer was ignored for the contentions. Two phases of the aggregates and the mortar matrix were considered when calculating the homogeneous Young’s modulus, $E_{ho}$, using the volume fraction, elastic modulus, and Poisson’s ratio of each phase. $E_{ho}$ can be expressed using Equation (21):

(21) $$E_{ho}=E_{m}+\frac{V_{p}\left(E_{a}-E_{m}\right)}{1+\left(1-V_{p}\right)\frac{E_{a}-E_{m}}{E_{m}+\frac{4\zeta_{m}}{3}}}=30+\frac{0.3\left(48-30\right)}{1+\left(1-0.3\right)\frac{48-30}{30+\frac{4\zeta_{m}}{3}}}=34.25\;\mathrm{GPa}$$

(22) $$\zeta_{m}=\frac{E_{m}}{2\left(1+\nu_{m}\right)}=\frac{30}{2\left(1+0.2\right)}=12.5\;\mathrm{GPa}$$

where $E_{m}$ is the Young’s modulus of mortar. $E_{m}$ is the Young’s modulus of aggregates. $V_{p}$ is the volume fraction of aggregates. $\nu_{m}$ is the Poisson’s ratio of mortar.

Based on the parameters in Table 3, the value of $E_{ho}$ was calculated to be 34.25 GPa by Equation (21). Compared to the value of the numerical result, 34.25 GPa was almost close to the value of 34.5 GPa assumed in the simulations.

#### 3.1.2. Nonlinear Mechanical Behaviors

The nominal stress–strain curves presented significant nonlinear mechanical behavior following the linear elasticity, as shown in Figure 12. The compressive stress had a short stage of slight oscillation following the linear increment of the curve. Then, the stress kept increasing with increased deformation until the maximum stress was reached. After the maximum of the curve, the concrete specimen lost its loading resistance, and the stress decreased sharply.

### 3.2. Failure Profiles

**Figure 13:**
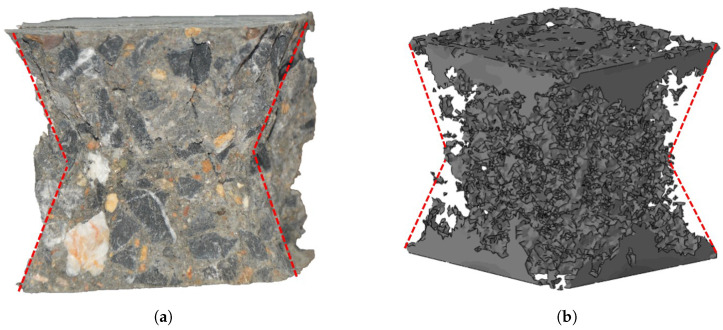
The final profiles of specimens showed a “sand-glass” (indicated as the red line) shape in both numerical and experimental results. (**a**) Final profiles of the experiment. (**b**) Final profiles of the meso-FE model.

A comparison of the final failure pattern of the cubic specimens was made between the experimental observations and the meso-scale FE results, as shown in Figure 13. Figure 13a shows the experimental result, in which a sand-glass-shaped column was found to form after removing the weakened fracture pieces on the cube’s surface. The fracture profiles showed that the damage was more serious in the middle than that at the upper and bottom ends. The meso-scale FE model also presented similar failure profiles, as shown in Figure 13b. In the numerical results, a sand-glass-shaped column could also be observed after removing the elements whose damage scale reached 1 (*d* = 1). This column typically formed due to friction between the loading plates and the contacted boundaries of the cube. The presence of contact friction influenced not only the failure profiles, but also the mechanical behavior of the system. The impact of friction will be elaborated upon in detail in the following section.

**Figure 14:**
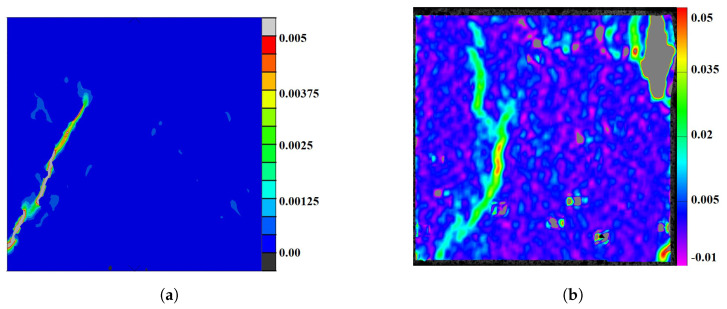
The first cracking profile that led to the nonlinear response of the stress–strain curves. (**a**) Compressive strain = 0.001 in the simulation. (**b**) Compressive strain 0.001 in the experiment.

The initial cracking profile exhibited a strong correlation between the experimental and simulated results, as illustrated in Figure 14. The first crack occurred at the nominal compressive strain of about 0.001 when the curve transited from a linear state to a nonlinear one in the experiment. Figure 14a shows the tensile strain map of the simulation, where an inclined tensile crack was formed near the left edge. Similar fracture profiles were observed in the experimental results of the tensile strain calculated from the DIC, where an inclined crack was formed near the left edge. The difference was that a small crack was observed at the upper-right corner, as shown in Figure 14b. Thus, the nonlinear behavior of the compressive stress–strain curves must be related to the meso-scale damage and cracking.

## 4. Discussion

### 4.1. Evolution of Compressing Failure

**Figure 15:**
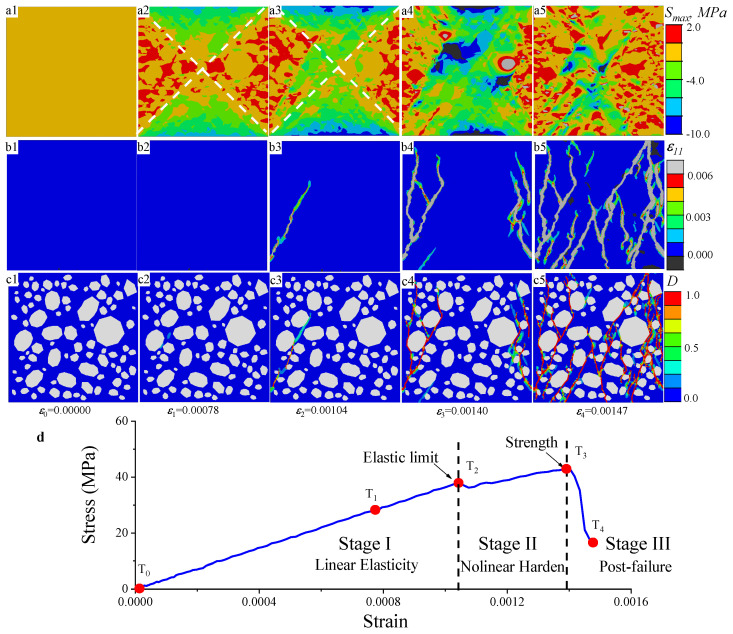
The evolution of the maximum principal stress (**a1**–**a5**), maximum principal strain (**b1**–**b5**), and damage maps (**c1**–**c5**) under the condition of uniaxial compression, where the numbers 1 to 5 are related to the points of the macro-stress–strain curve in (**d**).

Using the present meso-scale FE model, the failure process of concrete was simulated at the meso-scale under a uniaxial compression condition. Figure 15 describes the development of the maximum principal stress, the maximum principal strain, and the damage maps of concrete under a uniaxial compression at different loading stages. Note that the maps were taken from the slicingfrom the center of the cube. With the loads increasing, the distribution of the maximum principal stress was found to be a “sand-glass” shape, as shown in Figure 15(a1–a5). In most of the area of this “sand-glass”, the stress was negative, meaning a compression state. However, the area outside of this “sand-glass” showed a tensile stress state. As is known, tensile failure stress is commonly less significant than the compressive strength of brittle materials, contributing to the formation of the initial crack at the tensile triangular zones outside of the “sand-glass”. Through careful observation, the earliest damage merged at $T_{1}$, which was very close to the time when the compressive stress reached the peak, as shown in Figure 15(c2). Then, the first crack was formed and grew obliquely to the center of the cube according to the stress state of the “sand-glass”, as shown in Figure 15(b3). However, the compressive stress increased with increasing deformation after a slight reduction from $T_{2}$ to $T_{3}$, as shown in Figure 15d. During this period, more cracks emerged at the tensile triangular zones outside of the “sand-glass”. After the peak stress of $T_{3}$, the stress–strain curves showed a stress collapse from $T_{3}$ to $T_{4}$. Accordingly, several cracks could be found at the center of the cube, as shown in Figure 15(b5).

Therefore, the compression stress–strain curves could be divided into three stages according to the failure characteristics: the linear elastic stage (Stage I), the nonlinear hardening stage (Stage II), and the post-failure (Stage III). In Stage I, all components were kept at the linear elastic state without any crack observed. The maximum stress in Stage I can be defined as the elastic limit. In this stage, the stress dropped first and then kept nonlinearly strain-hardening until the maximum was reached, which could be defined as the strength of this test. Though the number of cracks increased with the increasing loading, the resistant center column could still bear the compression stress. Until Stage III, the cracks crossed through the center column, and the stress collapsed. In Stage III, the numerical smeared cracks resulted in stress loss. The formation of the final “sand-glass” profile was influenced by the stress distribution within the concrete cube. Notably, the peak stress was not attained when the initial crack appeared. However, once cracks began to develop at the center of the column, the stress reached its peak, causing the concrete cube to lose its compressive resistance capacity.

### 4.2. Effect of Friction

**Figure 16:**
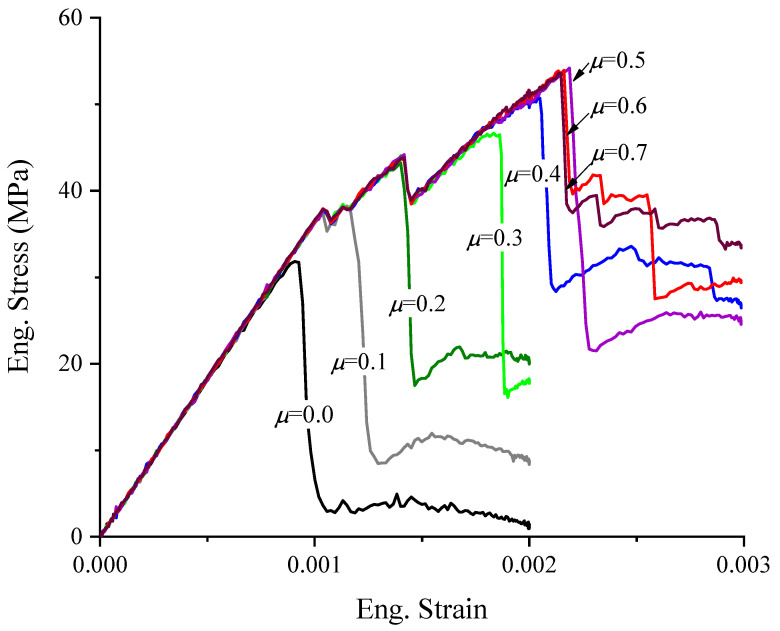
The compressive stress–strain curves of different contacting friction properties with the change of friction coefficients ranging from 0.0 to 0.7.

A “sand-glass” related to the friction between the rigid loading plates of universal test machines and the contacted surfaces of cubes has been previously reported [20,22]. For instance, Hao [34] conducted friction tests and obtained a real friction coefficient between concrete and steel of 0.235. Zhang [20] set the friction coefficient between the specimen and steel plate as 0.1 in a simulation work, utilizing similar experimental materials and loading conditions as those adopted by Hao and Zhang. Thus, μ = 0.2 can be thought to be close to the real friction coefficient. To further investigate the effect of friction, a series of friction coefficients was set for the contacting surfaces between the rigid loading plates and cubic specimens. Previous researchers [35] have reported the friction coefficient between concrete and steel plates to range from 0.1 to 0.7 in experiments. Thus, eight friction coefficients ranging from 0.0 to 0.7 were set as the contacting properties to discuss the effect of friction on the meso-scale FE model. Figure 16 shows eight compressive stress–strain curves under different contacting conditions with the changes in the friction coefficients ranging from 0.0 to 0.7.

**Figure 17:**
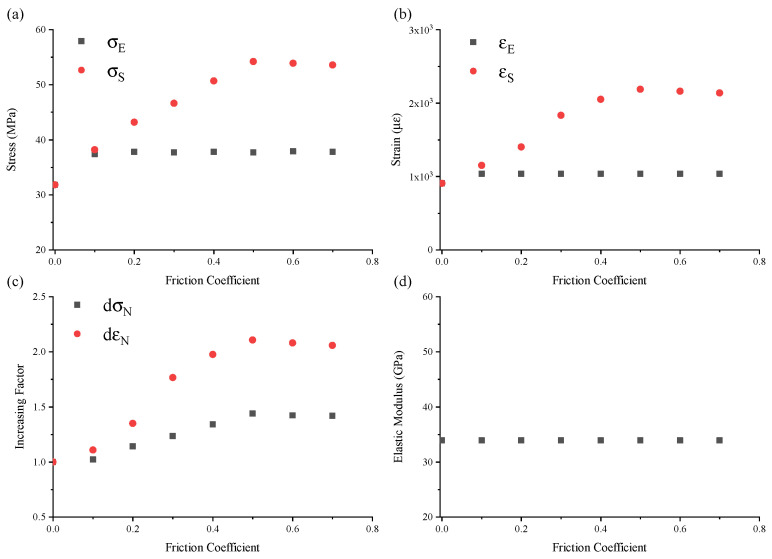
Characteristic parameters with changes in friction coefficients ranging from 0.0 to 0.7: elastic limit, $\sigma_{E}$, strength, $\sigma_{S}$ (**a**), normalized stress or strain increment (**b**), Δσ or Δε (**c**), and average elastic modulus, *E* (**d**).

As shown in Figure 15d, the effect of friction on the mechanical behavior was discussed as the division of three stages: linear elasticity, nonlinear hardening, and post failure. Herein, several parameters summarized in Figure 17 were taken to characterize the effect of friction on the mechanical responses of concrete under uniaxial compression: the elastic limit, $\sigma_{E}$, strength, $\sigma_{S}$, normalized stress or strain increment, $d\sigma_{N}$ or $d\varepsilon_{N}$, and average elastic modulus, *E*. Note that the average elastic modulus, *E*, is the slope of the linear elastic stage of the engineering stress–engineering strain curve before the elastic limit in Figure 14. The normalized stress or strain increment, $d\sigma_{N}$ or $d\varepsilon_{N}$, can be expressed as follows:

(23) $$\left\{\begin{array}{c} d\sigma_{N}=\frac{\sigma_{S}}{\sigma_{E}}\\ d\varepsilon_{N}=\frac{\varepsilon_{S}}{\varepsilon_{E}} \end{array}\right.$$

In Stage I, the elastic modulus almost stayed constant at 33.9 GPa with the change of μ, as shown in Figure 15. However, a difference could be observed between the curve μ = 0 and μ > 0 within the elastic limits. The elastic limit with μ > 0 was larger than that with μ = 0, which could also stay constant with increasing μ when μ > 0. Actually, μ should commonly be >0, as it is quite difficult to conduct any test with μ = 0. Thus, one may conclude that contacting friction had a rather limited influence on the elastic mechanical behavior of the concrete in the experiments.

**Figure 18:**
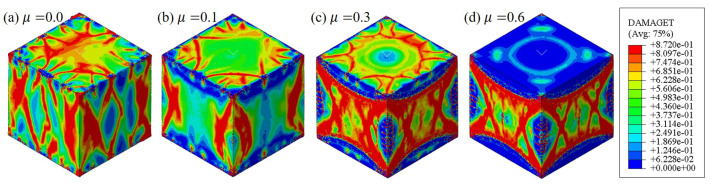
The final failure profiles with different friction coefficients shown in the damage cloud map.

In Stage II, the nonlinear hardening behavior led to a large difference in the compressive stress–strain curves, as shown in Figure 16. Firstly, significant nonlinear hardening behavior occurred due to the stress when μ≥0.1. The concrete lost resistance capacity as soon as the elastic limit was reached as the stress–strain curve μ=0. Due to the strength $\sigma_{S}$ with an increasing μ when μ≤0.5, even a significant linear increase was observed. Interestingly, $\sigma_{S}$ seemed to stop the increasing trend and even decreased slightly when μ> 0.5. The strain of $\varepsilon_{S}$ corresponding to $\sigma_{E}$ and $\sigma_{S}$ showed the same trend with an increasing μ, as Figure 17b shows. Secondly, with an increasing μ, the nonlinear hardening was more significant. The increment of the strain $d\varepsilon_{N}$ was larger than that of the stress $d\sigma_{N}$ in this stage, as shown in Figure 17c. Thirdly, multiple oscillations were observed in Stage II in the numerical results when μ≥0.1, and the number of oscillations increased with an increasing μ until μ=0.5, as shown in Figure 16. Actually, the oscillations were related to the emergence and propagation of cracks, as described in Section 4.1. After exceeding the elastic limit, numerical micro-cracks were gradually formed and spread with the increasing compressive deformation of the macro-cubic specimens. As this progressed, the friction confined the micro-cracks from growing to macro-cracks close to the contacting surface of the specimen, and the confinement would be stronger with increasing μ. Thus, an increase occurred due to the strength with the increase of μ when μ< 0.5. However, the strength stopped increasing when μ≥ 0.5, which might be related to the confinement caused by the contacting friction reaching the stress, which was large enough to suppress the formation of micro-cracks at the contacting surface. Figure 18 shows the final failure profile of the specimens after compression with different μ values. In Figure 18, the damage in Figure 18d at the contacting surface is clearly less than the damage profiles in Figure 18a–c. Only a small area of the contacting surface showed apparent damage, most of which presented, respectively, low damage and even no damage, as shown in Figure 18d. This result potentially explains the cessation of the strength increase when μ≥ 0.5.

In Stage III, the resistant columns could be found, as shown in Figure 18. The “sand-glass” shape can be seen more clearly with an increasing μ after the peak stress. Thus, the contacting friction between the loading plates and cubic specimens had a significant influence on both the mechanical performance and the failure profiles.

## 5. Conclusions

In this study, a well-designed 3D meso-FE model was employed to investigate the effect of friction on the mechanical behavior and failure characteristics of concrete under uniaxial compression loading. The effect of friction was discussed by changing the contacting friction coefficients of a simulation model from 0.0 to 0.7, which was demonstrated to be effective for the above mechanical behavior and failure profiles of the simulation and matched well with the experimental results. Based on this model, the following was found:

(1) The contacting friction had quite a small influence on the compression elastic mechanical behavior of the concrete;
(2) The nonlinear hardening behavior of the stress–strain curves had a quite strong relationship with the contacting friction;
(3) The final failure profiles of the experiments showed a “sand-glass” shape, which may be expected to result from the contacting friction;
(4) The contacting friction had a significant influence on both the mechanical performance and the failure profiles of the concrete.

Despite a well-designed 3D meso-FE model applied in this study to investigate the effect of friction, the weakness remains that the constitutive model and parameters were not discussed in depth. Herein, the constitutive model of aggregates was assumed to be elastic without considering the scenario of failure. However, this may lead to the overestimation of the strength and failure performance. In the future, the failure of the aggregate should be taken into consideration for the meso-scale FE simulation.

## Figures and Tables

**Table 1:** Size distribution of aggregates according to a classic Fuller curve [24].

Sieve Size (mm)	Total Percentage Retained (%)	Total Percentage Passing (%)
12.7	0	100
9.5	23	77
4.75	74	26
2.36	100	0

**Table 2:** The grain number of aggregates calculated according to a classic Fuller curve.

Grain diameter (mm)	9.5–12.7	4.75–9.5	2.36–4.75
Grain number	19	57	268

**Table 3:** Constitution parameters of the three phases in concrete.

Composition	Elastic Modulus (GPa)	Poisson’s Ratio	Compressive Strength (MPa)	Tensile Strength (MPa)	Fracture Energy (N/m)	Stiffness MPa/mm
Aggregate	48	0.2	-	-	-	-
Mortar	30	0.2	50.2	3.8	100	-
ITZ	30	0.2	33.6	2.66	120	$6\times 10^{5}$

**Table 4:** Element-related information of mortar, aggregate, and ITZ.

Component	Element Type	Element Size (mm)	Number
Mortar	C3D4H	1	964,611
Aggregate	C3D4H	1	182,557
ITZ layer	Zero-thickness cohesive elements	1	57,808

## Data Availability

Data are contained within the article.

## Abbreviations

The following abbreviations are used in this manuscript:

3-D	three-dimensional
DIC	digital image correlation
FE	finite-element
ITZ	interface transition zone
*d*	diameter of each grading class
$d_{c}$	compression scalar stiffness degradation variable
$d_{max}$	maximum aggregate diameter
$d_{min}$	minimum aggregate diameter
$d_{t}$	tensile scalar stiffness degradation variable
*D*	scalar stiffness degradation variable
$E_{c}$	Young’s modules of the weaker concrete
$E_{ho}$	homogeneous Young’s modulus
$E_{m}$	Young’s modules of the mortar
${E^{pl}}_{0}$	initial elastic modulus
*G*	flow potential
$G_{c}$	Kirchhoff’s modules of the weaker concrete
$G_{f}$	fracture energy
*K*	stiffness matrix
$K_{c}$	ratio of the second stress invariant of the tensile meridian to that of the compressive meridian
μ	frictional coefficient
*n*	exponent of the chosen grading curve
*N*	amount of aggregates
$\bar{p}$	effective hydro-static pressure
*P*	loading force
P(d)	corresponding passing percentage
$\bar{q}$	Mises equivalent effective stress
$q_{cm}$	compression meridian
$q_{tm}$	tensile meridian
*t*	nominal traction stress vector
$T_{0}$	original thickness of the cohesive element
$T_{eff}$	effective traction
$U_{t0}$	cracking displacement
*V*	volume of the concrete sample
$V_{e}$	equivalent volume of a single aggregate
$v_{P}$	volume fraction of aggregates
$w_{P}$	total weight of aggregate particles
α	characteristic parameter of yield function, α
β	characteristic parameter of yield function, β
γ	characteristic parameter of yield function, γ
δ	separation
ε	strain
$\varepsilon^{pl}$	plastic strain
ϵ	parameter, referred to as the eccentricity of materials
ψ	dilation angle
ν	Poisson’s ratio
$V_{p}$	specific weight
σ	Stress
$\bar{\sigma }$	effective stress
$\sigma_{b0}$	initial equi-biaxial compressive yield stress
$\sigma_{c0}$	initial uniaxial compressive yield stress
$\sigma_{cf}$	compression failure stress
$\sigma_{cy}$	yield stress,
$\sigma_{t0}$	tension strength
$\sigma_{tf}$	tension failure stress
ς	module parameter of mortar
